# Short course palliative radiotherapy in advanced solid tumors: a pooled analysis (the SHARON project)

**DOI:** 10.1038/s41598-022-25602-7

**Published:** 2022-12-05

**Authors:** Costanza Maria Donati, Gabriella Macchia, Giambattista Siepe, Alice Zamagni, Anna Benini, Francesco Cellini, Milly Buwenge, Savino Cilla, Silvia Cammelli, Stefania Rizzo, Luciana Caravatta, Tigeneh Wondemagegnhu, A. F. M. Kamal Uddin, Biniyam Tefera Deressa, Mostafa A. Sumon, Elisa Lodi Rizzini, Alberto Bazzocchi, Alessio G. Morganti, Francesco Deodato, Eleonora Farina

**Affiliations:** 1 Radiation Oncology, Department of Experimental, Diagnostic and Specialty Medicine-DIMES, Alma Mater Studiorum University of Bologna, Via Albertoni 15, 40138 Bologna, Italy; 2 IRCCS Azienda Ospedaliero-Universitaria Di Bologna, Bologna, Italy; 3 Dipartimento di Scienze Radiologiche ed Ematologiche, Università Cattolica del Sacro Cuore, Rome, Italy; 4 Radiation Oncology Unit, Gemelli Molise Hospital - Università Cattolica del Sacro Cuore, Campobasso, Italy; 5 Università Cattolica del Sacro Cuore, Dipartimento Universitario Diagnostica per immagini, Radioterapia Oncologica ed Ematologia, Rome, Italy; 6 Istituto di Radiologia, Università Cattolica Sacro Cuore, Rome, Italy; 7 Medical Physics Unit, Gemelli Molise Hospital-Università Cattolica del Sacro Cuore, Campobasso, Italy; 8 Service of Radiology, Imaging Institute of Southern Switzerland, Ente Ospedaliero Cantonale (EOC), Lugano, Switzerland; 9 Department of Radiation Oncology, Santissima Annunziata Hospital, Gabriele D’Annunzio University of Chieti-Pescara, Chieti, Italy; 10 Radiotherapy Department, Tikur Anbessa Specialized Hospital, Department of Clinical Oncology, College of Medicine and Health Sciences, University of Gondar, 9086 Addis Ababa, Ethiopia; 11 Department of Radiation Oncology, United Hospital Limited, Dhaka, Bangladesh; 12 Diagnostic and Interventional Radiology, IRCCS Istituto Ortopedico Rizzoli, Bologna, Italy; 13 Radiotherapy Unit, Azienda Ospedaliero-Universitaria Di Ferrara, Ferrara, Italy

**Keywords:** Cancer, Medical research, Oncology, Signs and symptoms, Physics

## Abstract

Previous trials showed the tolerability and efficacy of a palliative radiotherapy (RT) regimen (SHARON) based on the 4 fractions delivered in 2 days in different oncological settings. In order to identify possible predictors of symptomatic response, the purpose of this study is to perform a pooled analysis of previous trials. We analyzed the impact on symptomatic response of the following parameters: tumor site, histological type, performance status (ECOG), dominant symptom, and RT dose using the Chi-square test and Fisher’s exact test. One-hundred-eighty patients were analyzed. Median RT dose was 20 Gy (range: 14–20 Gy). The overall response rate was 88.8% (95% CI 83.3–92.7%) while pre- and post-treatment mean VAS was 5.3 (± 7.7) and 2.2 (± 2.2), respectively (*p* < 0.001). The overall response rate of pain, dyspnea, bleeding, dysphagia, and other symptoms was 86.2%, 90.9%, 100%, 87.5%, and 100%, respectively. Comparing the symptomatic effect based on the analyzed parameters no significant differences were recorded. However, patients with locally advanced disease showed a higher rate of symptomatic responses than metastatic ones (97.3% vs 83.0%; *p* = 0.021). Finally, the complete pain response rate was more than double in patients with mild to moderate (VAS: 4–7) compared to those with severe (VAS > 7) pain (36.0% vs 14.3%; *p* = 0.028). This pooled analysis showed high efficacy of the SHARON regimen in the relief of several cancer-related symptoms. The markedly and significantly higher complete pain response rate, in patients with mild-moderate pain, suggests early referral to palliative RT for patients with cancer-related pain.

## Introduction

Radiation therapy (RT) has a marked effect on tumor-related symptoms. Therefore, palliative RT is frequently used in the treatment of symptomatic patients with advanced cancer.

Short treatments are preferable in the setting of palliative RT. In fact, the poor patients’ clinical conditions limit the use of prolonged therapies. In addition, the symptomatic relief from even low RT doses, and therefore the low risk of toxicity, promotes the use of accelerated treatments delivered in few fractions (accelerated-hypofractionated RT). Finally, the treatment acceleration is theoretically associated with a more rapid tumor response and therefore with better and faster palliative response.

Some studies tested regimens based on four fractions administered in two days in the palliative treatment of advanced tumors of the uterine cervix^1,2^ and of the head and neck^3–5^. These trials showed good tolerability and symptomatic efficacy using doses of approximately 14–15 Gy in two days and repeating these treatments up to three times 2–4 weeks apart.

We tested a similar regimen (SHARON: SHort-course Accelerated radiatiON therapy) but in a single cycle, in the same and other settings (multiple brain and complicated bone metastases, advanced tumors of head-neck, thorax, and pelvis). A first series of phase I trials demonstrated the feasibility, using 3D-conformal RT, of administering 18–20 Gy in two days^6,7^. Furthermore, subsequent phase II trials recorded consistently higher than 80% response rates in the settings mentioned above^8,9^.

However, our studies evaluated the SHARON regimen in single settings and in small patients series and therefore some questions remain open. In particular, it is not known whether SHARON is equally effective regardless of the anatomical site of the treated lesion, histological type, patient's performance status, and the dominant symptom. Furthermore, the impact of moderate doses (< 18 Gy) versus high doses (18–20 Gy) on symptom relief was not previously analyzed. Therefore, the purpose of this study is to perform a pooled analysis of the previous trials, on a large patients population, in order to clarify these topics.

## Materials and methods

### Study design and end points

This is a retrospective pooled analysis of symptomatic response in patients with locally advanced or metastatic tumors undergoing the SHARON regimen and enrolled in prospective phase I and II trials. The methods used for RT planning and delivery, patient evaluation and data analysis were previously described^6–9^ and will only be summarized here.

### Inclusion criteria

Patients with symptomatic locally advanced or metastatic solid tumors were included in the SHARON trials. Patients with ECOG performance status > 3, pregnant, aged < 18 years, and previously irradiated in the same anatomic site were excluded.

### Radiotherapy planning and delivery

RT was planned with a CT-simulation in the treatment position. The gross tumor volume (GTV) was defined as the symptomatic macroscopic lesion. The clinical target volume (CTV) was defined as the GTV with the addition of 1.0 cm isotropic margin while the planning target volume was defined as the CTV *plus* an additional 1.0 cm isotropic margin. The latter margin was 1.5 cm in the cranial-caudal direction in patients with thoracic or upper abdomen tumors. The dose prescription and specification was based on the ICRU 62 report^10^. The two daily RT fractions were delivered with an interval of 6 h. However, in patients with spinal cord irradiation, the interval was extended to 8 h. Before each fraction, a set-up check was performed using an electronic portal imaging device as previously described^11^.

### Follow up

Patient evaluation based on physical examination and complete blood count, was performed 15 days after RT. During the same visit we evaluated both symptomatic response and quality of life using the Cancer Linear Analogue Scales (CLAS), a tool based on an analog-visual scale, with values from 0 to 10, measuring the level of well-being (CLAS1), fatigue (CLAS2), and the ability to carry out daily activities (CLAS3)^12^. Subsequently, patients were evaluated every two months by recording symptoms’ control and RT-related toxicity. Further instrumental tests were required based on the physician's clinical judgment.

### Clinical and statistical issues

Pain was scored both using a Visual Analogue self-assessment Scale (VAS) and according to the International Atomic Energy Agency criteria, using the pain score and drug score parameters^13^. Pain response was graded according to International Bone Metastases Consensus Working Party criteria^14^. For other symptoms, the patient's reported reduction or complete disappearance of symptoms was defined as partial and complete response, respectively. Furthermore, the complete suspension of ongoing therapies for the specific symptom was required to define a complete response. The overall response rate was defined as the sum of complete and partial responses. The symptomatic progression-free actuarial survival curve during follow-up was calculated with the Kaplan–Meier method. Toxicity was scored according to the Radiation Therapy Oncology Group (RTOG) and the RTOG/European Organization for Research and Treatment of Cancer criteria for acute and late adverse events, respectively^15^. We used the chi-square test of independence, or Fisher’s Exact test for contingency tables in case of a cell-count of less than 5, to analyze the impact of the considered categorical parameters on symptomatic response. Statistical analyses were performed using the IBM SPSS Statistic version 20 software package.

### Ethical issues

All trials on which this analysis is based were approved by the Ethics Committees of the institution in which the enrollment and treatment were performed (Gemelli Molise Hospital, Campobasso, Italy). All experiments were performed in accordance with relevant guidelines and regulations. All patients signed a written consent to their inclusion in the trials before RT planning.

### Ethics approval and consent to participate

The study was approved by the local Ethics Committee. All patients provided written informed consent before the study entry.

## Results

**Figure 1:**
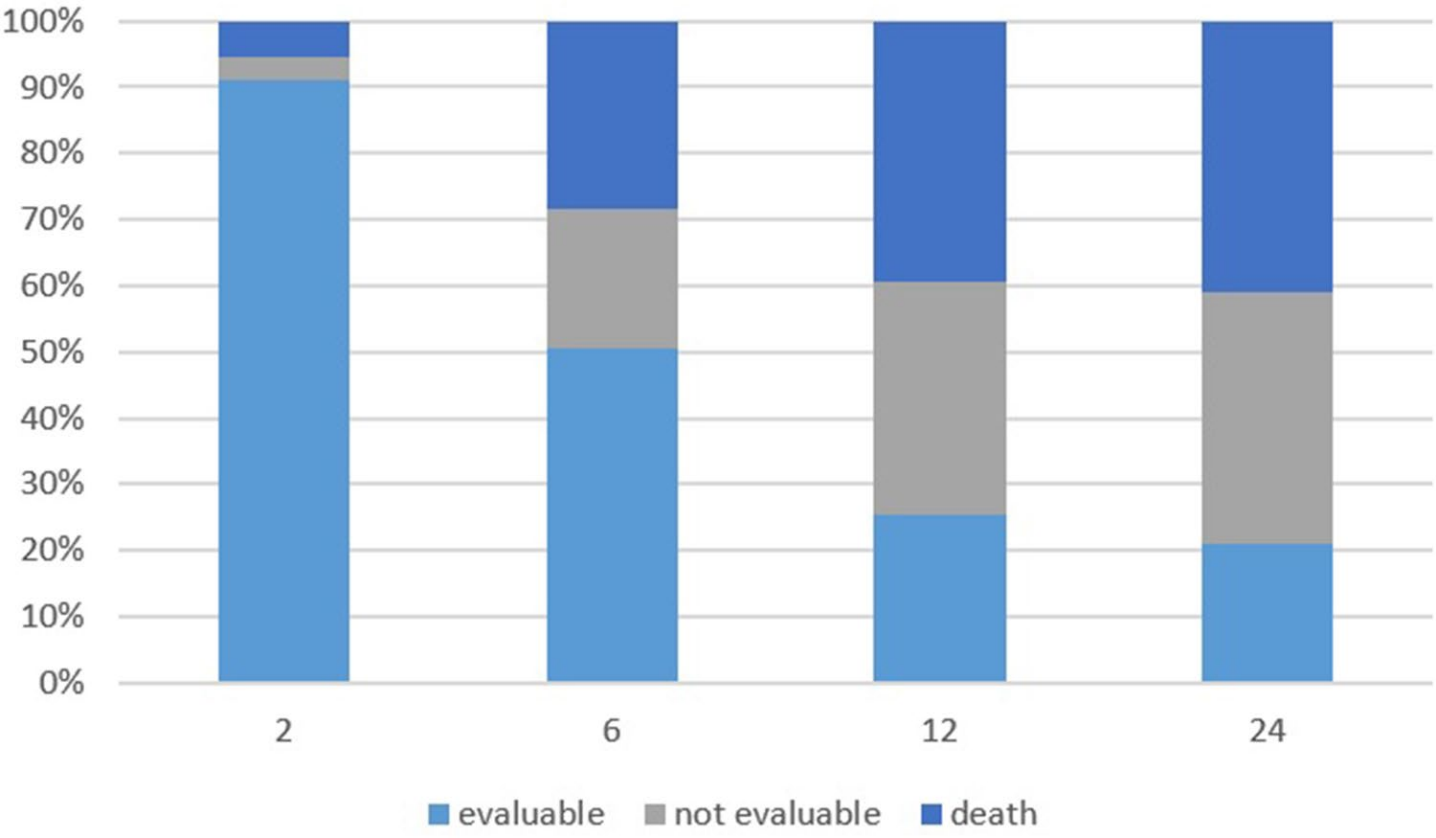
Drop-out trend during the study period.

One hundred and eighty patients were included in this pooled analysis (M/F: 100/80; median age: 70 years; range: 39–98 years; median follow-up: 5 months; range: 1–36 months). Other characteristics of the subjects included in this study are shown in Table 1. No patient died before the evaluation 15 days after radiotherapy, and all patients were examined at the first follow-up after treatment (15 days). Instead, at the first visit of follow-up (2 months) the rate of patients who died or were not evaluable, due to worsening of the general condition that precluded the visit, was 6% and 3%, respectively. Moreover, the rate of patients who could be evaluated at 6 and 12 months was 50% and 25%, respectively (Fig. 1).

**Table 1:** Symptomatic response and univariate analysis.

	n	Complete symptoms remission		Partial symptoms remission		No change		Symptoms progression		Overall response rate*		*p*	Fisher’s exact test
		n	%	n	%	n	%	n	%	n	%		
**Tumor stage**													
Advanced primary cancer	75	24	32.0	49	65.3	2	2.7	0	0	73	97.3	0.021	0.017
Metastatic cancer	105	27	26.0	60	57.0	12	11.5	6	5.5	87	83.0		
**Histologic type**													
Squamous cell carcinoma	62	22	35.5	35	56.5	3	4.8	2	3.2	57	92.0	0.766	0.750
Adenocarcinoma	74	18	24.3	47	63.5	7	9.5	2	2.7	65	87.8		
Others	44	11	25.0	27	61.3	4	9.0	2	4.7	38	86.3		
**Site**													
Head and neck	52	13	25.0	31	59.6	6	11.5	2	3.9	44	84.6	0.179	0.214
Thorax	54	14	25.9	38	70.4	2	3.7	0	0	52	96.3		
Bone	49	17	34.7	23	46.9	5	10.2	4	8.2	40	81.6		
Pelvis	25	7	28.0	17	68.0	1	4.0	0	0	24	96.0		
**ECOG**													
0–1	80	23	28.8	47	58.8	7	8.8	3	3.6	70	87.6	0.953	0.949
2–3	100	28	28.0	62	62.0	7	7.0	3	3.0	90	90.0		
**Baseline symptoms**													
Pain	116	31	26.7	69	59.5	10	8.6	6	5.2	100	86.2	0.524	0.593
Dyspnea	22	4	18.2	16	72.7	2	9.1	0	0	20	90.9		
Bleeding	11	5	45.5	6	54.5	0	0	0	0	11	100		
Dysphagia	8	0	0	7	87.5	1	12.5	0	0	7	87.5		
Other	9	4	44.4	5	55.6	0	0	0	0	9	100		
Multisymptomatic	14	7	50.0	6	42.9	1	7.1	0	0	13	92.9		
**Radiotherapy dose**													
< 18 Gy	36	6	16.7	27	75.0	2	5.6	1	2.7	33	91.7	0.257	0.248
18–20 Gy	144	45	31.2	82	57.0	12	8.5	5	3.3	127	88.2		

ECOG-PS: Eastern Cooperative Oncology Group-Performance Status.

*Complete *plus* partial remission.

**Figure 2:**
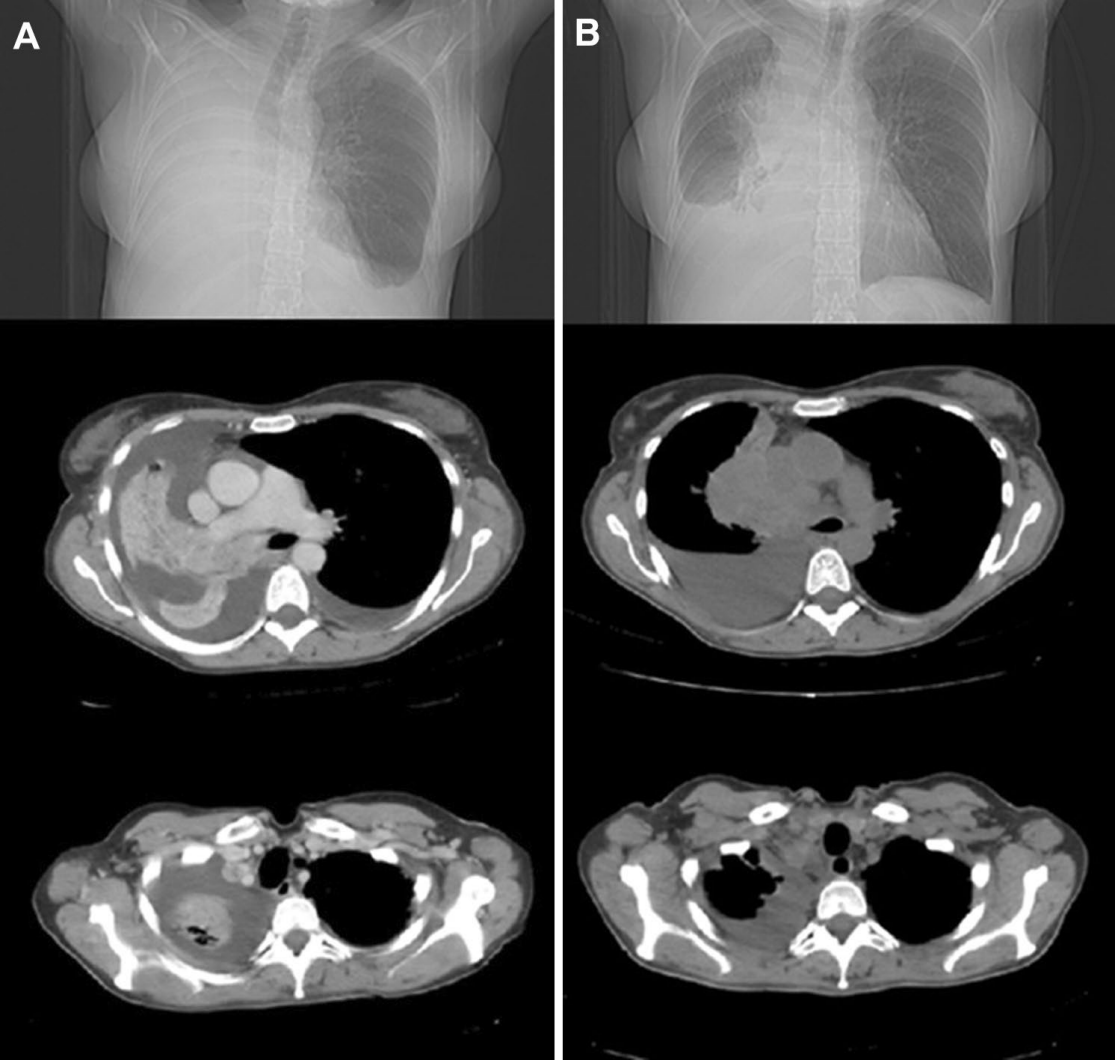
Chest X-ray and CT scan of a patient with locally advanced non-small cell lung tumor before (**A**) and after (**B**) accelerated-hypofractionated radiotherapy (20 Gy in four fractions). The patient reported complete response of dyspnea three days after the treatment.

Figure 2 shows the radiological response of an advanced non-small cell lung tumor after palliative RT. A complete or partial symptomatic response was recorded in 51 (28.3%) and 109 (60.5%) patients, respectively, with 88.8% (95% CI 83.3–92.7%) overall response rate and four months median time to symptoms progression. The pre- and post-treatment mean VAS was 5.3 (± 7.7) and 2.2 (± 2.2) respectively (*p* < 0.00001). At first follow-up, 76 (42.2%), 84 (46.7%), and 20 (11.1%) patients showed improved, stable, and worse ECOG performance status, respectively. An improvement of CLAS 1, 2, and 3 scores was recorded in 80 (48.2%), 70 (42.2%), and 77 (46.4%) out of 167 evaluable patients, respectively. Four patients (2.2%) presented Grade ≥ 3 acute toxicity while one patient (0.6%) showed Grade 3 late toxicity. Comparing the symptomatic effect of the SHARON regimen between anatomical sites, histological types, ECOG scores and RT doses (< 18 vs ≥ 18 Gy) no significant differences were recorded (data not shown). Similarly, the response rate of the different symptoms was similar, without significant differences (data not shown). In contrast, patients with locally advanced disease showed a higher rate of symptomatic responses than metastatic ones (97.3% vs 83.0%; *p* = 0.021). Finally, the complete pain response rate was more than double in patients with mild to moderate (VAS: 4–7) pain compared to those with severe (VAS > 7) pain (36.0% vs 14.3%; *p* = 0.028).

## Discussion

With 88.8% overall symptomatic response rate and only five patients (2.8%) with severe toxicity, this pooled analysis confirmed the tolerability and efficacy of the SHARON regimen in the relief of cancer-related symptoms. In particular, the analysis showed a symptomatic effect independent of tumor site, histological type, prevalent symptom, and delivered RT dose. However, even without statistically significant differences, it can be noted that bleeding showed the highest overall response rate (100%) and that squamous cell tumors showed a slightly higher response rate than adenocarcinomas (92.0% vs 87.8%). Furthermore, our analysis did not show a significant impact of RT dose on symptom relief. This result may be important because it suggests the use of this regimen (with possibly reduced doses) even in settings where advanced or simply conformal RT techniques are not available or in case of reirradiation.

In contrast, a higher response rate of the SHARON regimen was observed in patients with locally advanced disease than in those with metastatic tumors. It is not easy to explain this difference. It can be assumed that metastatic patients have generally multiple lesions with difficult registration of the symptomatic effect in the irradiated lesion alone.

Furthermore, our analysis showed a significantly higher complete pain response rate in patients with mild to moderate pain compared to patients with severe pain. This result suggests that palliative RT is less effective in terms of pain relief in patients with more severe symptoms, as confirmed by a previous analysis showing a higher rate of pain palliation in patients not receiving opioid therapy^16^. Furthermore, based on another analysis showing that long-lasting oncologic pain is significantly worse^17^, it can be hypothesized that early palliative RT may be more effective than RT delivered to subjects with more prolonged pain. This hypothesis would be in line with a further recent analysis showing that patients treated with palliative RT and with shorter pain duration (< 1 month) had higher cumulative incidence of pain response (subdistribution hazard ratio, 2.43; 95% CI 1.35–4.38) compared with subjects with longer pain duration (≥ 4 months)^18^. However, we should also consider that the higher efficacy of early palliative RT could derive from a milder pain in the initial stages of its onset but also from the well-known refractoriness of long-lasting and therefore chronic pain.

As anticipated, previous studies tested regimens based on four fractions in two days, with a total dose lower than ours but repeating RT up to three consecutive cycles. It is not possible to compare their results with those of our analysis because they used accelerated regimens in single tumor settings (mainly cervix and head and neck)^3–5^. However, the uniformly high response rates we recorded with only one RT course cast doubt on the need to treat all patients with repeated cycles. In fact, an alternative and more convenient strategy could be based on the delivery of a single course in patients with satisfactory symptomatic response and reserving repeated treatments only for non-responders.

This analysis has obvious limitations. A relatively basic Patients Reported Outcome Measure (CLAS scale) was used to assess quality of life. Furthermore, the retrospective design of the study is an additional limitation, even if the patients were enrolled, treated, and analyzed prospectively.

Despite these limitations, some clinical conclusions can be drawn. In fact, based on its tolerability and efficacy, the SHARON regimen can be proposed in clinical practice especially in: (i) patients residing far from the RT center; to minimize the need for travel and overnight stays, (ii) busy RT departments, in order to reduce waiting lists and to allow prompt treatment of symptoms, (iii) in situations such as the current SARS-CoV-2 pandemic, where it is preferable to limit the stay of cancer patients in the hospital environment due to the risk of infection.

Moreover, these results justify the design of further studies to improve the effectiveness of this regimen. In fact, despite uniformly high overall response rates, the complete symptomatic response rate was < 50% in all analyzed subgroups. Therefore, new trials could aim to: (i) improve the current results in terms of tumor response with the use of innovative irradiation modalities such as Partially Ablative RT (PART)^19^ and techniques based on the use of microbeams (GRID, lattice)^20^; (ii) further improve the toxicity profile through the use of modulated RT techniques^5^; (iii) optimize the integration between palliative RT and pharmacological palliative care; (iv) test the SHARON regimen in developing countries, where this short treatment would be particularly useful given the shortcomings in terms of RT centers and equipment^21^.

In conclusion, this pooled analysis showed the symptomatic efficacy of the SHARON regimen independent of anatomic site, histological type, performance status, prevalent symptom, and delivered RT dose. Seven randomized trials are currently underway in our centers to compare the results of this accelerated treatment (20 Gy in two days) with those of a traditional palliative RT treatment (30 Gy in 2 weeks) in different tumor settings (NCT03804307, NCT03804333, NCT03503682).

## Data Availability

The datasets used and/or analysed during the current study available from the corresponding author on reasonable request.
